# Retrospective, multicentre evaluation of central congenital hypothyroidism in the UK

**DOI:** 10.1530/ETJ-26-0014

**Published:** 2026-05-15

**Authors:** Catherine Peters, Claire Wood, James M Law, Chloe Stevens, Fatemah Alhusaini, Darla Rigby, Hannah Hornby, Tim Cheetham, Nadia Schoenmakers

**Affiliations:** ^1^ Great Ormond Street Hospital for Children, London, UK; ^2^ Translational and Clinical Research Institute, Newcastle University, Newcastle upon Tyne, UK; ^3^ Great North Children’s Hospital, Newcastle upon Tyne, UK; ^4^ University of Nottingham, Nottingham, UK; ^5^ Nottingham University Hospitals NHS Trust, Nottingham, UK; ^6^ Department of Metabolism and Systems Science, College of Medicine and Health, University of Birmingham UK, Birmingham, UK; ^7^ Institute of Metabolic Science, University of Cambridge, Cambridge, UK

**Keywords:** congenital hypothyroidism, congenital hypopituitarism, neonatal screening, central hypothyroidism

## Abstract

**Objective:**

Central congenital hypothyroidism (incidence ∼1:13,000) occurs in isolation (40% cases) or with additional pituitary hormone deficiencies. T4 ± TSH-based neonatal screening detects central congenital hypothyroidism within the first two weeks of life, permitting prompt treatment, but the UK TSH-based screening programme will not detect these cases. We delineated clinical characteristics, time-frame and pathway to diagnosis in clinically diagnosed individuals.

**Methods:**

Records were reviewed for 118 cases diagnosed from 1996 to 2022, in four tertiary centres.

**Results:**

Median age at diagnosis was 68 days (range: 1–5,056). 96% had combined pituitary hormone deficiencies. Non-specific neonatal concerns (hypoglycaemia/jaundice/weight concerns, 83%) and significant neurodevelopmental defects (34%) occurred frequently. Compared with cases diagnosed late (>1 year, *n* = 42), early diagnosis (≤14 days *n* = 23) was associated with neonatal intensive care admission (78 vs 29%, *P* < 0.001) and ACTH deficiency (96 vs 40% *P* < 0.0001). Mean FT4 was moderately low at diagnosis (−2.7 ± 0.9 SDS), but initial thyroid function was within reported reference ranges in 31 cases. Treatment delays could be substantial, even following detection of subnormal FT4, especially in late-diagnosed cases (mean: 208 ± 486 days).

**Conclusion:**

UK central congenital hypothyroidism cases are diagnosed later than screening-detected cases, and isolated TSH deficiency may evade detection entirely. ‘Sicker’ neonates are diagnosed earlier, but late diagnosis frequently occurs despite neonatal/childhood morbidity attributable to combined pituitary hormone deficiencies. Challenges include non-specific neonatal signs, requirement for bespoke age-specific FT4 reference ranges, lack of biomarkers for alternative diagnoses and masking by concomitant GH deficiency. Our findings mandate further studies to assess practicalities, costs and justification for introducing UK-wide central congenital hypothyroidism screening.

## Introduction

Primary congenital hypothyroidism (CHT) is the commonest neonatal endocrine disorder, occurring due to defective thyroid development or hormonogenesis (UK permanent CHT incidence ∼1:2,000) (1). In contrast, central congenital hypothyroidism (CeCHT) is rare (estimated maximal incidence 1:13,000) and attributable to hypothalamic or pituitary dysfunction, resulting in decreased thyroid-stimulating hormone (TSH) stimulation of the thyroid gland. CeCHT may occur in isolation, but most (60–80% cases) occur in association with multiple pituitary hormone deficiencies (MPHD) (2, 3, 4). Delayed treatment of neonatal hypothyroidism may be associated with irreversible neurodevelopmental delay; therefore, CHT is screened for in developed countries to facilitate prompt diagnosis. However, the UK neonatal CHT screening programme uses a TSH-based protocol, which will successfully detect primary CHT, but not CeCHT in which TSH is usually inappropriately normal or low (5). Worldwide, a minority of screening programmes measure total (TT4) or free T4 (FT4) and TSH simultaneously or stepwise, permitting detection of both primary CHT and CeCHT (2). Recent European guidance on central hypothyroidism (6) and European guidelines on congenital hypothyroidism (7) recommend consideration of neonatal screening strategies capable of detecting central congenital hypothyroidism. However, UK-specific data describing the phenotype and timing of diagnosis of clinically detected cases remain limited.

The inclusion of total T4 in the CHT neonatal screening programme remains a topic of debate. Historically, arguments against screening for CeCHT include its rarity and the perception that it is usually milder than primary CHT and the belief that it will be identified in early life because of the manifestations of other hormone deficiencies. Moreover, T4-based programmes that detect CeCHT may have lower sensitivity for detecting primary CHT. However, data from the Netherlands, which operates an unique screening algorithm based on combined measurement of TSH, T4 and thyroxine-binding globulin (TBG), have shown that CeCHT may be more severe than previously anticipated, in addition to having a comparable incidence to other conditions currently included in the screening programme (e.g. phenylketonuria) (8, 9). Since CeCHT is usually associated with combined pituitary hormone deficits, detection of CeCHT on screening may also facilitate prompt diagnosis and treatment of growth hormone (GH) and ACTH deficiency before affected individuals present clinically with potentially life-threatening features, e.g. hypoglycaemia (10). Moreover, testing for mini-puberty following a neonatal diagnosis of CeCHT has the potential to permit optimal early management of gonadotrophin deficiency, which may have an impact on future reproductive function (11), although evidence of long-term impact on fertility is currently limited. However, even well-established CeCHT programmes detect high numbers of false positives, mandating continuous re-evaluation and modification to improve specificity, although this is partially mitigated by exclusion of low TT4 due to TBG deficiency (12, 13, 14). False-positive results are particularly recognized in association with physiological immaturity and non-thyroidal illness, and this has informed screening strategies in some programmes, including restriction of TT4-based screening in preterm infants in the Netherlands (4, 6, 7, 15).

In the UK, since CeCHT is not included in the CHT neonatal screening programme, cases are diagnosed following clinical presentation. This has hampered the calculation of the incidence of CeCHT, and the efficiency of clinical diagnosis has not been formally assessed. We performed a retrospective evaluation of case notes from 118 individuals diagnosed with CeCHT and managed in four UK tertiary centres in order to delineate the clinical characteristics, outcomes and pathways to diagnosis in a UK CeCHT cohort.

## Methods

### Ethical approval

Initial data retrieval was performed at individual centres as part of trust-registered audits or service evaluation projects. Amalgamation of data and subsequent analyses were performed under the auspices of an ethically approved project (REC 23/NE/0137) with approvals from local R&D departments, with informed consent not required due to the preservation of anonymity and the non-interventional nature of the study.

### Case selection criteria

Participating centres comprised four tertiary centres in England: Great Ormond Street Hospital, London (GOSH); Newcastle Hospitals NHS Foundation Trust, Newcastle upon Tyne (NH); Nottingham University Hospitals NHS Trust, Nottingham (NUH); and Cambridge University Hospitals NHS Foundation Trust, Cambridge (CUH). Each centre provides a paediatric endocrine service with expertise in managing CeCHT. Site-specific data were compiled by the local teams i) through central retrieval of case details via local audit departments using ICD codes and/or ii) departmentally, using databases specific to each paediatric endocrine team (Supplementary Data (see section on Supplementary materials given at the end of the article)).

Notes were reviewed manually, and patients were included if they had a secure diagnosis of CeCHT and adequate available clinical and biochemical data. Manual filtering resulted in the inclusion of *n* = 118 cases (CUH: *n* = 18, GOSH: *n* = 52, NH: *n* = 29 and NUH: *n* = 19) for further analysis.

### Data collection

Clinical, radiological and biochemical data were retrieved retrospectively according to a standardized questionnaire by a local clinician with expertise in paediatric endocrinology. This captured the time-frame and clinical and biochemical pathway to diagnosis and treatment of CeCHT, with further details regarding data collection and definitions provided in Supplementary Data. Family history was not systematically recorded as part of this retrospective chart review. Categorical responses were recorded as ‘yes’ or ‘no’ if there were robust supporting data. Otherwise, ‘not known’ (NK) or ‘not applicable’ (NA) was used and responses were amalgamated for analyses.

### Data analysis

Analyses were performed to define the clinical and biochemical characteristics of CeCHT and to compute the time-frame to diagnosis and treatment. In addition to whole-cohort analyses, patients were stratified into three groups by age of diagnosis. Age cut-offs, although arbitrary, were chosen to define individuals diagnosed within the approximate time-frame in which neonatal screen-detected CeCHT would be detected: 0–14 days (Group 1: *n* = 23 cases), cases with unequivocally late diagnosis at greater than 1 year of age (Group 3: *n* = 42, including 22 school-age children) and those diagnosed within a broad time-frame in between (Group 2: 15–365 days, *n* = 51). Data were interrogated to identify potential challenges to diagnosis and management and to delineate areas of unmet need, including an assessment of the need for future studies to address the potential feasibility of screening for CeCHT in the UK. Two individuals were not included in age-stratified analyses as the precise age at diagnosis was unclear.

### Statistical analysis

Data were expressed as median and range or mean and SD (or SEM where stated). Group-wise comparisons were performed using Kruskal–Wallis ANOVA with Dunn’s post hoc test for multiple comparisons. Categorical variables were compared using Fisher’s exact test. Analysis software included GraphPad Prism, version 10.4.1, and StataSE, version 15.1. *P* values <0.05 were taken as indicators of statistical significance.

## Results

### Cohort characteristics

A total of 118 cases (79 males and 39 females) were evaluated, of whom 96% (*n* = 113) had multiple pituitary hormone deficiencies (MPHD) and 5 (4%) had isolated TSH deficiency. Diagnosis of CeCHT was achieved at a median age of 68 days (range: 1–5,056). All age-stratified groups demonstrated a similar slight excess of males over females. Additional anterior pituitary hormone deficits included GH deficiency (109 cases, 92%) and ACTH deficiency (83 cases, 70%). Gonadotrophin deficiency was confirmed in 46 cases (39% overall, 46% of males) but not determined for 29 cases (25%, 19% of males), usually due to the young age or female sex of participants. Posterior pituitary dysfunction due to vasopressin deficiency was evident in 10 cases (8%) (Table 1).

**Figure 1:**
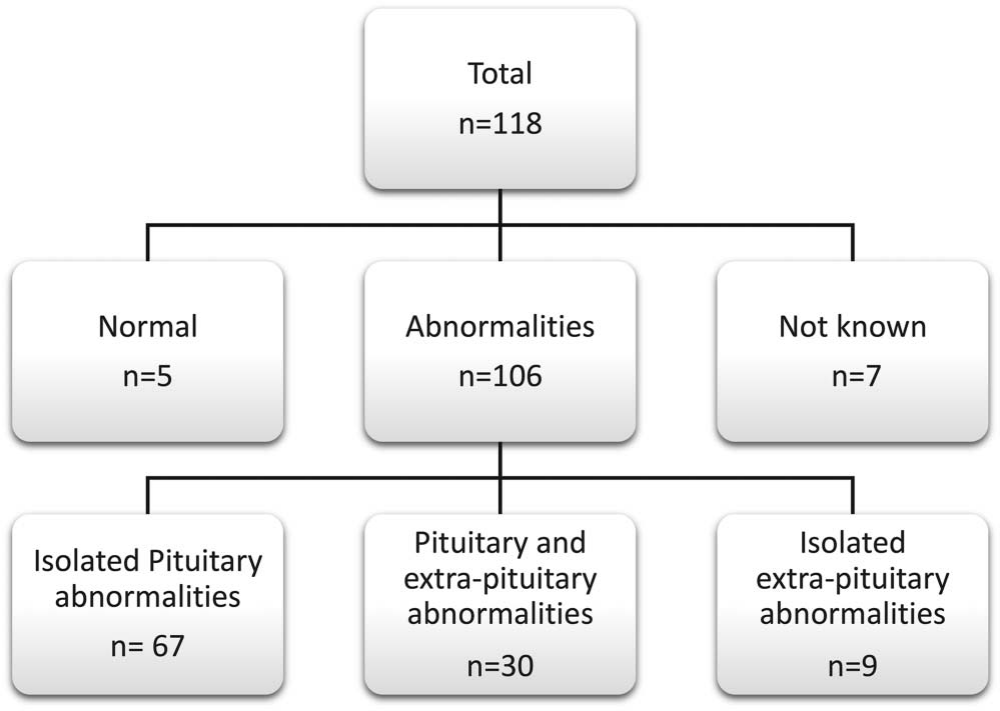
Diagram summarizing clinically reported magnetic resonance imaging (MRI) or computerized tomography (CT) findings across the cohort. *n* = number of cases.

Pituitary imaging (MRI or CT) was available in 111 cases and demonstrated abnormalities in 95% (*n* = 106). Analysis was limited to the clinical radiological report, which was variably detailed. Anatomical abnormalities were localized to the pituitary in 60% (*n* = 67), and extra-pituitary abnormalities were noted in 35% (*n* = 39), most frequently including septo-optic dysplasia or optic nerve hypoplasia (*n* = 19, 49% cases with extra-pituitary involvement). Only 5 cases (5%) were reported as having normal imaging (Fig. 1).

Isolated TSH deficiency most commonly has an identifiable genetic basis; however, only one individual with isolated CeCHT had undergone screening of known causative genes and harboured a hemizygous *IGSF1* mutation. It was unclear how many cases with MPHD had had targeted screening of genes associated with congenital hypopituitarism, but causative mutations were recorded for six cases (*POU1F1*:* n* = 3, *PROP1*:* n* = 1, *GLI2*:* n* = 1 and *OTX2*:* n* = 1). Copy number variations of uncertain significance were identified in three cases, and two individuals had Prader–Willi syndrome (including one with isolated TSH deficiency).

### Clinical features

**Figure 2:**
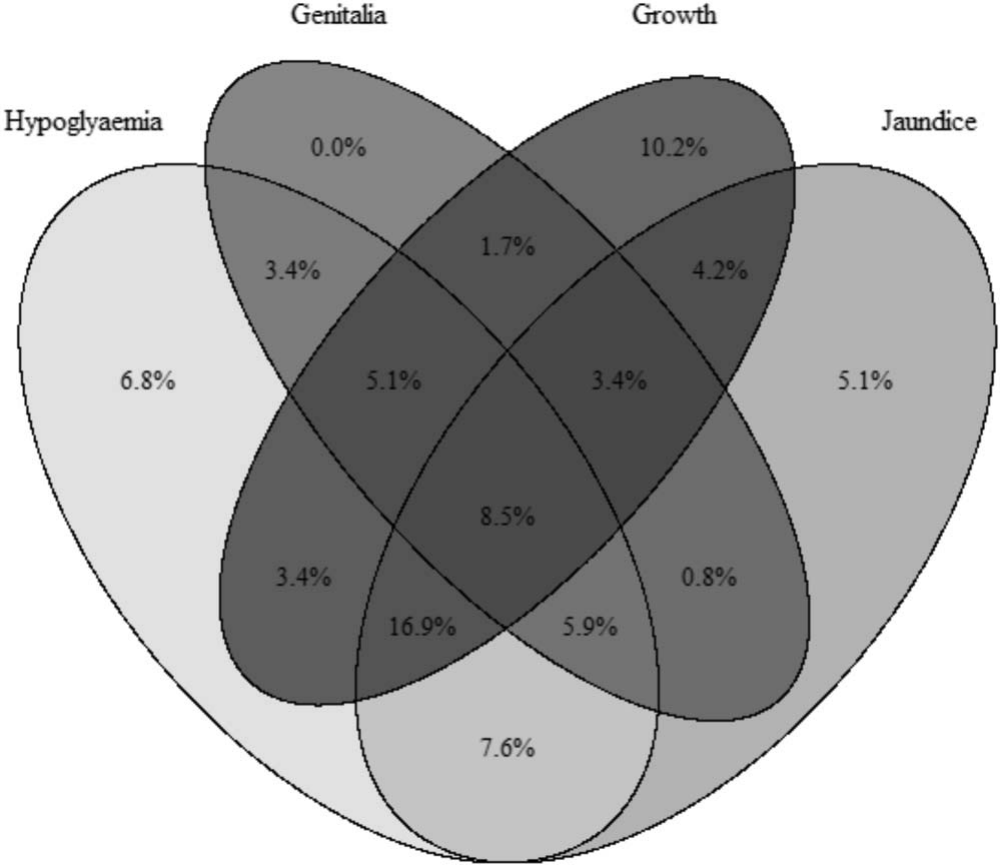
Venn diagram illustrating clinical features of hypopituitarism, and their concurrence, across the cohort. Numbers refer to the percentage of the cohort. Genitalia: abnormal genitalia. Growth: neonatal growth concerns. Hypoglycaemia and jaundice: neonatal occurrence of these signs.

Cases were screened for neonatal and childhood clinical features supporting a diagnosis of hypopituitarism (summarized in Tables 1 and 2 and Fig. 2). Overall, neonatal clinical concerns were frequent throughout the cohort with 59 cases (50%; unknown for 33 cases, 28%) requiring admission to the neonatal intensive care unit (NICU) although only 23 cases (19%) were born preterm (preterm status unknown for 30 cases, 25%). Non-specific neonatal signs were common, with 53% cases exhibiting jaundice (34% unknown), 58% reported to have neonatal hypoglycaemia (21% unknown) and documented concerns about perinatal weight noted in 53% (18% unknown). Mean weight z score at diagnosis was −1.8 ± 1.9. At least one of the following neonatal features was recorded in 87% of cases: NICU admission, jaundice, perinatal weight concerns or hypoglycaemia, although available data did not permit accurate cohort-wide delineation of their severity or duration. Of 79 males, 42% exhibited clinical features of hypogonadism (29% of overall cohort).

**Table 1:** Clinical and endocrine features of evaluated cases. Data are presented as mean ± SEM or as *n* (%). Cases for whom data were unavailable are termed ‘unknown’. Ages at diagnosis were 0–14 days (group 1), 15 days–1 year (group 2) and >1 year (group 3). *P* values (*P < 0.05, **P < 0.01, ***P < 0.001) were calculated using Fisher’s exact test for categorical variables and Kruskal–Wallis test with Dunn’s post hoc test for continuous variables. Significance was defined as a *P* value < 0.05.

Parameters	Group 1	Group 2	Group 3	*P* value
Total	23	51	42	
Sex				NS
Male	19 (83%)	33 (65%)	26 (62%)	
Female	4 (17%)	18 (35%)	16 (38%)	
Preterm birth				*
Yes	3 (13%)	16 (31%)	4 (10%)	
Unknown	4 (17%)	12 (24%)	13 (31%)	
NICU admission				***
Yes	18 (78%)	28 (55%)	12 (29%)	
Unknown	4 (17%)	15 (29%)	13 (31%)	
Weight Z-score				NS
At diagnosis	−1.3 ± 0.5	−2.1 ± 0.3	−1.8 ± 0.4	
Unknown	11 (48%)	27 (53%)	19 (45%)	
Pituitary hormone deficiencies				
GH	22 (96%)	45 (88%)	40 (95%)	NS
ACTH	22 (96%)	42 (82%)	17 (40%)	***
Gonadotrophin				NS
Yes	14 (61%)	18 (35%)	14 (33%)	
Unknown	3 (13%)	17 (33%)	8 (19%)	

ACTH, adrenocorticotrophic hormone; GH, growth hormone; NICU, neonatal intensive care unit; NS, not significant.

**Table 2:** Clinical features of hypopituitarism in evaluated cases. Data are presented as mean ± SEM with range in brackets for the number of neonatal signs, or the number of cases with percentage of the relevant group in brackets. Cases for whom data were unavailable are termed ‘unknown’. *P* values (*P < 0.05, **P < 0.01, ***P < 0.001) were calculated using Fisher’s exact test for categorical variables and Kruskal–Wallis test with Dunn’s post hoc test for continuous variables. Significance was defined as a *P* value < 0.05. Clinical signs of hypogonadism (cryptorchidism, pathologically small phallus) were documented for males only (*n* stated in each group). ‘Neonatal signs’ included jaundice, hypoglycaemia and perinatal growth concerns.

Parameter	Group 1	Group 2	Group 3	*P* value
Neonatal signs of HPT				
Hypoglycaemia				***
Yes	21 (91%)	34 (67%)	12 (29%)	
Unknown	1(4%)	8 (16%)	15 (36%)	
Jaundice				**
Yes	15 (65%)	32 (63%)	14 (33%)	
Unknown	5 (22%)	17 (33%)	17 (40%)	
Males, total *n*	19	33	26	NS
Males with clinical signs of hypogonadism	10 (53%)	15 (45%)	8 (31%)	
Unknown^†^	3 (16%)	5 (15%)	7 (27%)	
Neonatal weight concerns				*
Yes	8 (35%)	30 (59%)	25 (60%)	
Unknown	3 (13%)	7 (14%)	10 (24%)	
Number of neonatal signs				NS
Mean	2.0 ± 0.2	2.0 ± 0.1	1.6 ± 0.2	
Range	1–3	0–3	0–3	
HYG + ACTH deficiency	21 (100%)	32 (94%)	6 (50%)	***
Childhood signs of HPT				
Abnormal growth				**
Yes	8 (35%)	24 (47%)	31 (74%)	
Unknown	8 (35%)	21(41%)	10 (24%)	

HPT, hypopituitarism; HYG, hypoglycaemia; NS, not significant.

^†^ This row presents number of males for whom data regarding clinical signs of hypogonadism is unknown.

Individuals diagnosed earlier tended to be more unwell. In particular, the requirement for NICU admission was most common in cases diagnosed in the first 14 days of life (78%) and least common in those diagnosed late (29%), which was not explained by differences in preterm birth. Cases diagnosed earlier were also more likely to have neonatal hypoglycaemia or jaundice. Some of these features may be explained by the fact that ACTH deficiency was more common in the cases diagnosed at day 0–14 (96%) compared with those diagnosed at older ages (40% cases diagnosed at >1 year of age). Cases with neonatal hypoglycaemia diagnosed at ≤1 year almost invariably had ACTH deficiency, but only 50% of those diagnosed >1 year of age with a past history of neonatal hypoglycaemia were ACTH-deficient. Other hormone deficiencies, including GH deficiency, which was almost universal across the cohort, were similar between age-stratified groups, and there was no difference in cranial imaging findings between groups. Cases diagnosed at older ages were more likely to have had perinatal growth concerns or abnormal growth in childhood (Tables 1 and 2).

### Thyroid biochemistry

**Figure 3:**
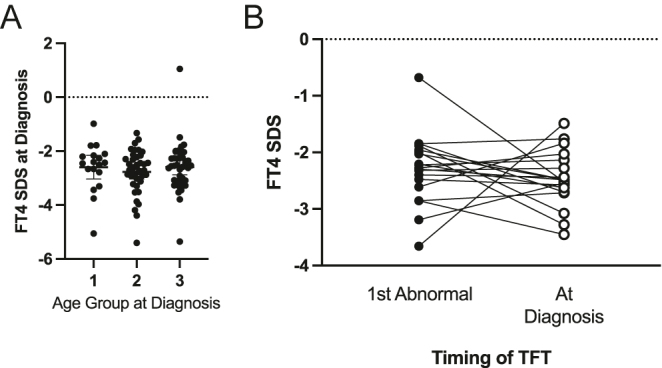
(A) FT4 SDS at diagnosis for each age group at diagnosis. Ages at diagnosis were 0–14 days (group 1), 15 days–1 year (group 2) and >1 year (group 3). *P* values were calculated using a Kruskal–Wallis test with Dunn’s post hoc test, but no values reached significance (defined as a *P* value < 0.05). The graph shows mean and 95% confidence intervals. (B) FT4 SDS at first abnormal thyroid function (1st abnormal) and at treatment initiation (at diagnosis) for cases diagnosed at >1 year of age for whom there was a delay in diagnosis of ≥30 days following first abnormal TFT (*n* = 16, SDS at both time points available for *n* = 13). FT4, free T4; TFT, thyroid function test.

Analysis of thyroid biochemistry is summarized in Table 3 and Fig. 3. Median FT4 at diagnosis was 8.8 pmol/L (range: 3.2–17.1); however, to accommodate the use of different assays and reference ranges in different centres, FT4 was subsequently expressed as standard deviation score relative to the local reference range. Median FT4 SDS at diagnosis was −2.6 (range: −5.4–1.1). The severity of CeCHT was consistent across age-stratified groups and thus did not explain the differing ages at diagnosis (Fig. 3A).

**Table 3:** Thyroid biochemistry at diagnosis. Data are presented as mean ± SEM or as *n* (%). Cases for whom data were unavailable are termed ‘unknown’. *P* values (*P < 0.05, **P < 0.01, ***P < 0.001) were calculated using Fisher’s exact test for categorical variables and Kruskal–Wallis test with Dunn’s post hoc test for continuous variables. Significance was defined as a *P* value < 0.05.

Parameters	Group 1	Group 2	Group 3	*P* value
Age, days				NA
Age at 1st TFT	8.7 ± 1	54 ± 9.5	1,203 ± 178	
Age at 1st abnormal TFT	8.3 ± 0.9	70.5 ± 11.9	1,684 ± 221	
Age at diagnosis	10.1 ± 0.8	84.9 ± 12.6	1812 ± 191	
Thyroid biochemistry				
TSH SDS at 1st abnormal TFT	0.4 ± 0.5	1.4 ± 0.4	0.4 ± 0.3	NS
FT4 SDS at 1st abnormal TFT	−2.5 ± 0.2	−2.6 ± 0.2	−2.5 ± 0.2	NS
FT4 SDS at L-T4 start	−2.6 ± 0.2	−2.8 ± 0.1	−2.6 ± 0.1	NS
Abnormal TFT at 1st check				***
Yes	16 (70%)	40 (78%)	18 (43%)	
Unknown	4 (17%)	3 (6%)	4 (10%)	
Time between 1st TFT and 1st abnormal TFT, days	0.4 ± 0.3	16 ± 7.6	449 ± 107	***
Group 1 vs group 3				**
Group 2 vs group 3				***
Time between 1st abnormal TFT and L-T4 commencement, days	1.4 ± 0.6	10.9 ± 3	208 ± 79	NS

TFT, thyroid function test; FT4, free T4; L-T4, levothyroxine; NA, not applicable (these parameters are strongly determined by group definitions); NS, not significant.

### Contributors to diagnostic delay

Delays in the diagnosis of CeCHT were attributable to three main factors. First, age at initial assessment of thyroid biochemistry following neonatal screening was highly variable (median age: 35 days, range: 0–4,500) and often occurred late despite the high prevalence of neonatal clinical concerns.

**Figure 4:**
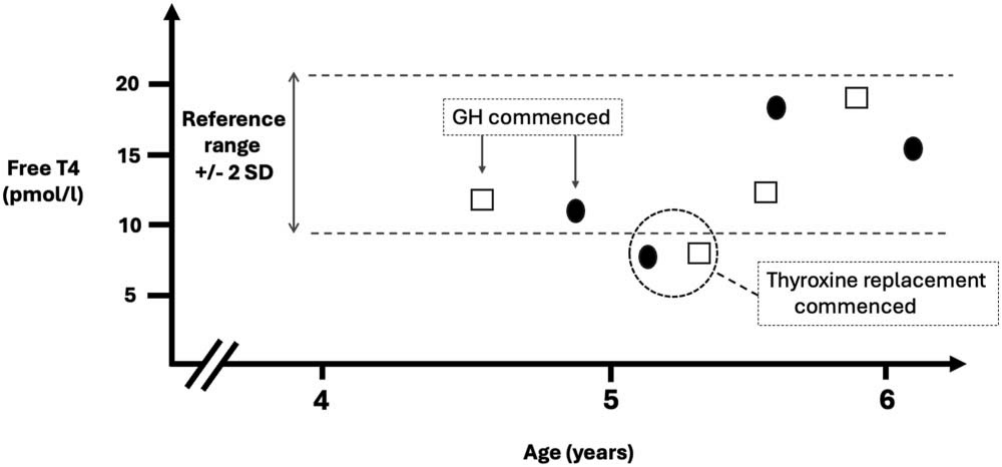
Secondary hypothyroidism manifest biochemically following the initiation of growth hormone therapy in two patients from one of the participating centres. Both had abnormal pituitary anatomy on MR imaging, including an ectopic posterior pituitary bright spot, and were short at the time of growth hormone (GH) initiation – patient 1 (open square) was 2.8 standard deviations (SD) below the mean for height and patient 2 (black oval) was 2.7 standard deviations below the mean for height. Both patients had measurable FT4 within the age-related reference range before thyroxine treatment was commenced and then FT4 concentrations below the assay threshold afterwards.

Second, initial thyroid function tests (TFTs) were reportedly normal in 31 cases, most frequently in those diagnosed at >1 year of age (48%) in whom the mean delay between initial thyroid function tests and first abnormal thyroid function tests was substantial (449 ± 660 days) (Table 3). Although the underlying causes for this could not be interrogated in detail, many cases were diagnosed in centres in which a lack of age-specific local reference data resulted in comparison of paediatric FT4 levels with adult FT4 ranges. Additionally, we noted several cases who were initially diagnosed with GH deficiency and exhibited a decline in FT4 levels below the normal range once GH replacement was commenced (5 out of 29 cases in whom GH was started before levothyroxine, in 1 centre). Exemplar cases are shown in Fig. 4.

Finally, some cases exhibited delays in treatment initiation following the first abnormal thyroid function test. This delay seemed most marked in cases diagnosed at >1 year of age (mean: 208 days, range: 0–2,563). In some cases, we observed prolonged, borderline subnormal FT4 levels, on repeated measurements, suggesting difficulties in interpreting thyroid biochemistry in these cases or an unwillingness to attribute the results to CeCHT (Fig. 3B).

### Neurodevelopmental outcomes

Adverse neurodevelopmental outcomes were frequent with 40 cases (34%) exhibiting objectively significant developmental concerns, defined by the presence of a formal Education, Health and Care Plan, EHCP (a UK statement that formalizes the need for a tailored approach to care and education due to neurodevelopmental or behavioural concerns). The exact number of cases with concomitant visual impairment due to optic nerve hypoplasia could not be ascertained, but 18 cases with significant developmental concerns had isolated pituitary defects or normal MRI scans, without extra-pituitary abnormalities. Autism spectrum disorder (ASD) (27 cases, 23%) and attention deficit hyperactivity disorder (ADHD) (16 cases, 14%) were also frequently reported, and either subjective or objective developmental concerns were noted in 67 individuals (57%). Adverse neurodevelopmental outcomes were comparable across age-stratified groups and did not correlate with NICU admission, neonatal hypoglycaemia or ACTH deficiency (Tables 4 and 5). However, individuals with radiologically detected extra-pituitary abnormalities were more likely to have significant clinical neurodevelopmental defects (Table 5).

**Table 4:** Neurodevelopmental features in evaluated cases. Data are presented as *n* (%). Cases for whom data were unavailable are termed ‘unknown’. *P* values (*P < 0.05, **P < 0.01, ***P < 0.001) were calculated using Fisher’s exact test. Significance was defined as a *P* value < 0.05. Severe developmental delay was indicated by the presence of a formal EHCP or equivalent.

Parameters	Group 1	Group 2	Group 3	*P* value
Severe developmental delay				NS
Yes	8 (35%)	14 (27%)	16 (38%)	
Unknown	1 (4%)	7 (14%)	3 (7%)	
ASD				NS
Yes	4 (17%)	14 (27%)	8 (19%)	
Unknown	2 (9%)	3 (6%)	2 (5%)	
ADHD				NS
Yes	3 (13%)	9 (18%)	4 (10%)	
Unknown	2 (9%)	5 (10%)	2 (5%)	
Developmental delay				NS
Yes	8(35%)	25 (49%)	27 (64%)	
Unknown	4 (17%)	5 (10%)	1 (2%)	
≥1 developmental concern	11 (48%)	29 (57%)	28 (67%)	NS

EHCP, Education, Health and Care Plan; ASD, autism spectrum disorder; ADHD, attention deficit hyperactivity disorder.

**Table 5:** Clinical and endocrine features in evaluated cases associated with the presence of a formal EHCP. The number of cases with or without an EHCP is recorded for each outcome. Cases for whom data were unavailable are termed ‘unknown’. *P* values (*P < 0.05, **P < 0.01, ***P < 0.001) were calculated using Fisher’s exact test. Significance was defined as a *P* value < 0.05.

	EHCP in place			*P*-value
	Yes	No	Unknown	
NICU admission				NS
Yes	17	38	4	
No	10	15	1	
Unknown	13	14	6	
Neonatal hypoglycaemia				NS
Yes	23	40	5	
No	8	16	1	
Unknown	9	11	5	
ACTH deficiency				NS
Yes	30	47	6	
No	10	20	5	
Unknown	0	0	0	
Associated EPA				**
Yes	22	16	1	
No	18	47	7	
Unknown	0	4	3	

EPA, extra-pituitary abnormalities; EHCP, Education, Health and Care Plan.

## Discussion

This study is the first multicentre, large-scale evaluation of clinically diagnosed CeCHT cases in the UK. Previous evaluations of CeCHT have been performed in screening-detected populations in the Netherlands (3) and clinically diagnosed cohorts in the USA and Israel (42 and 94 cases, respectively) (16, 17). A smaller cohort of 54 clinically diagnosed UK cases was evaluated at Great Ormond Street Hospital over 20 years ago (18).

A disproportionate number of our cases have MPHD (96%) compared with 61% screened cases in the Netherlands, and similarly, increased frequencies of MPHD (84–98%) have been noted in other clinically diagnosed cohorts (3, 16, 17). Since up to 39% of screening-detected cases in the Netherlands have isolated TSH deficiency (8% transient), this raises concerns that isolated CeCHT evades clinical detection in the UK (3). This is well reported in IGSF1 deficiency, the commonest cause of isolated CeCHT, for which diagnosis in the proband frequently results in late identification of hitherto undiagnosed CeCHT across several generations. Several case reports have demonstrated adverse neurodevelopmental or growth sequelae due to delayed treatment in affected families, supporting a need for earlier diagnosis (19, 20). The majority of isolated CeCHT is genetically mediated, thus necessitating universal analysis of causative genes (*IGSF1, IRS4, TBL1X, TRHR* and *TSHB*); however, only one affected individual in our cohort had genetic testing despite UK-wide availability of diagnostic gene panels. This suggests under-utilization of genetic testing, and we would advocate protocolization of genetic evaluation to facilitate family screening as part of isolated CeCHT management in the future. The male predominance observed in this cohort may, in some cases, reflect undiagnosed X-linked IGSF1 deficiency, which can present with isolated TSH deficiency and may also be associated with additional pituitary hormone deficiencies (usually prolactin or growth hormone), potentially leading to misclassification as MPHD in the absence of genetic testing.

Hormone deficiencies in MPHD cases were similar to previously reported cohorts with almost universal occurrence of GH deficiency (92% vs 78–96% in other cohorts), followed by ACTH deficiency (70%, 81–86% in other series) and infrequent posterior pituitary dysfunction. The true prevalence of gonadotrophin deficiency was not evaluable due to the young age of many of the participants (3, 17). Hypothyroidism was comparable in severity to CeCHT in other cohorts, with mean FT4 at diagnosis 8.7 pmol/L (3, 17). The commonest MRI abnormalities associated with MPHD are reportedly pituitary stalk interruption syndrome (PSIS). Although the majority of our cohort had MRI abnormalities restricted to the pituitary, the variable quality of standard clinical reporting precluded detailed analysis (3).

CeCHT may be associated with adverse sequelae, such as neonatal jaundice and concerns regarding growth as well as hypoglycaemia in the presence of associated ACTH and /or GH deficiency. In keeping with cohort-wide deficiencies of GH and ACTH deficiency in addition to CeCHT, NICU admission, neonatal hypoglycaemia and jaundice occurred frequently across all age groups. Concerns regarding perinatal weight gain or childhood growth were predominantly noted in cases diagnosed later, reflecting the longer time required for these to manifest. Although GH deficiency was near-universal, hypoglycaemia was almost invariably associated with concomitant ACTH deficiency in cases diagnosed at ≤1 year of age, consistent with a causal link in this age group. Neonatal hypoglycaemia without ACTH-deficiency occurred in 50% cases diagnosed over one year of age supporting an additional, GH-specific contribution to glucose homeostasis.

The relatively late diagnosis of CeCHT across our cohort was not explained by differences in biochemical severity at age of diagnosis and was underpinned by three main factors. First, delayed recognition of clinical features of hypopituitarism frequently resulted in late evaluation of thyroid function (median age: 35 days, range: 0–4,500). Whilst this may seem surprising, comparable time-frames were noted in clinically diagnosed cases in Israel (median age at diagnosis: 50 days, range: 1–8,760, 34% diagnosed within the first 2 weeks) (17). Additionally, in screened CeCHT cases in the Netherlands, although 88% were hospitalized in the first weeks of life for feeding problems, hypoglycaemia or prolonged jaundice, 49% were discharged without a CeCHT diagnosis until the screening results were available (3). We were unable to evaluate whether children born more recently were diagnosed with CeCHT at younger ages, as these birth cohorts were younger overall.

Genital features of hypogonadism are highly suggestive of hypopituitarism and delayed diagnosis in this context indicates a need to improve awareness among clinicians reviewing affected cases. However, other signs (e.g. hypoglycaemia, jaundice and growth concerns) are poorly specific. This presents a major diagnostic challenge in CeCHT and MPHD and may explain why ‘sicker’ neonates benefitted from earlier diagnosis but 17% of cases reached school age before diagnosis. Neonatal hypoglycaemia (<2.6 mmol/L) affects approximately 40% of neonates, and hyperbilirubinaemia occurs in around 50%, usually without substantial underlying pathology. Persistent or recurrent neonatal hypoglycaemia and prolonged jaundice are more concerning, especially in combination (21, 22, 23). A limitation of our retrospective notes analysis was frequent reliance on clinical details reported in clinic letters, precluding accurate quantitation of the duration or biochemical severity of these events. Family history was not systematically recorded in this retrospective cohort, which may have limited assessment of inherited patterns of disease, particularly in cases of isolated TSH deficiency. Future prospective analyses may yield more specific diagnostic clues for CeCHT. In the absence of screening, there is a need both for improved awareness of the clinical presentations of CeCHT and for refinement of existing management guidelines for neonatal jaundice or hypoglycaemia, which do not consistently acknowledge hypopituitarism as a potential precipitant (23, 24, 25).

The second contributor to delayed diagnosis was the apparently normal initial thyroid biochemistry in 16 and 48% cases diagnosed in age groups 2 and 3 respectively. This is of particular concern when considering newborn screening for CeCHT, as a high penetrance of the condition in the neonatal period is a prerequisite for successful detection. Exact values were not available for these ‘normal’ thyroid hormone measurements in the majority of individuals, which is a limitation of the study. It is generally accepted that thyroid function tests in children should include FT4 in addition to TSH as standard. However, local practice varies, with a requirement for FT4 to be specifically requested as a separate test, in addition to TSH, in some units. Additionally, many UK centres do not use local paediatric age-specific reference ranges, despite this being a prerequisite for correct interpretation of FT4 levels, which are higher in healthy children than in adults. Subnormal FT4 levels may therefore have been inappropriately classified as ‘normal’ in many of these cases (26, 27). Additionally, we noted that children initially diagnosed with GH deficiency sometimes exhibited ‘unmasking’ of pre-existing CeCHT, following commencement of GH treatment likely due to associated alterations in thyroid hormone deiodination and release (28). Interpreting thyroid biochemistry in the context of overall pituitary status is therefore important. Such ‘unmasking’ of CeCHT may also occur in cases with early normal neonatal sampling if maternally transferred thyroid hormones were contributing to FT4 measurements (29).

We cannot exclude a contribution from genuine evolving TSH deficiency, particularly in MPHD due to *PROP-1* mutations or SOD (10, 18). Nonetheless, it is likely that CeCHT cases with truly normal neonatal T4 levels (measured on dried blood spots) or FT4 levels measured on separated venous serum samples are rare. Although two published series reported frequent normal neonatal T4 measurements in clinically diagnosed CeCHT cases, these results may have been artefactual due to low sensitivity of the fixed cut-offs used (16, 17). ‘Unmasking’ of CeCHT was noted in only 6.3% of children with apparent isolated GHD in a large Dutch series (30). Moreover, estimated numbers of cases missed on newborn screening in the Netherlands are low, approximating to 0.8 cases of MPHD and 0–2 cases of isolated CeCHT per year between 1995 and 2015 although the actual number may be higher (14).

The final barrier to timely diagnosis is the biochemical challenge of diagnosing central hypothyroidism (when measured FT4 is subnormal compared to an appropriate reference range), which has not been assessed in previous studies of clinically diagnosed cases. The mainstay of diagnosis comprises the association of low FT4 concentrations with inappropriately low or normal TSH concentrations. Circulating T3 levels are often normal, and in hypothalamic dysfunction, elevated immunoreactive TSH may be noted, albeit with subnormal bioactivity (10). This thyroid biochemistry has a broad differential diagnosis, of which non-thyroidal illness and transient hypothyroxinaemia of prematurity are particularly relevant in an unwell neonatal cohort (31). Current biomarkers cannot differentiate between these conditions, and the value of TRH testing is controversial (18, 32). Rarely, unrelated endogenous (e.g. thyroid hormone resistance alpha and Allan–Herndon–Dudley syndrome) or exogenous causes (e.g. medication, or the neonatal consequences of maternal thyroid dysfunction during pregnancy) may result in subnormal FT4 levels (33, 34). Biochemical ambiguity, together with hesitancy to commit a child to potentially life-long levothyroxine treatment, likely contributed to treatment delays in this cohort. However, the high pre-test probability of CeCHT should, in retrospect, have triggered earlier initiation of levothyroxine and these recurrent diagnostic challenges substantiate the need to develop improved biomarkers to distinguish true CeCHT from its biochemical mimics.

A key argument in favour of screening for CeCHT is perceived opportunity for early intervention to mitigate the adverse neurodevelopmental effects of untreated CeCHT. Despite a lack of controlled studies, screening-detected CeCHT cases with FT4 levels comparable to those causing cognitive deficits in primary CHT, demonstrate comparable full-scale intelligence quotient (FSIQ) with siblings following early treatment, supporting a beneficial effect of early diagnosis (35). Clinically detected CeCHT has a 37–50% rate of associated neurodevelopmental defects with the highest rate in MPHD, perhaps due to additional effects of hypoglycaemia or cerebral anatomical defects (16, 17, 35). In isolated CeCHT, neurodevelopmental defects can range from speech delay to profound intellectual impairment requiring special schooling, but even individuals with milder biochemical hypothyroidism may be affected (19, 20, 36). Neurodevelopmental concerns occurred frequently in our cohort especially in those with extra-pituitary anatomical abnormalities, and ASD and ADHD, which have not been investigated in previous series, were also found to be common, although a control population was not available for comparison. The absence of correlation between neurodevelopmental outcomes and age at diagnosis may reflect the retrospective design of the study and should not detract from the fact that this cohort is vulnerable to adverse neurodevelopmental features and requires prompt and optimal management of thyroid function.

A key driver for this work was the question of whether the UK should adapt its newborn screening programme to include screening for CeCHT. Conclusions must be caveated by the retrospective nature of the study and reliance on notes review, with unavailable data in some areas potentially affecting reliability. Nonetheless, the results highlight a clear need to improve diagnosis in CeCHT and demonstrate that affected individuals are vulnerable to neonatal and childhood morbidity, consistent with current European Thyroid Association guidance on central hypothyroidism and European congenital hypothyroidism guidelines, which recommend consideration of neonatal screening strategies capable of detecting central congenital hypothyroidism (6, 7). Moreover, there may be substantial under-detection of isolated TSH deficiency. If, as expected, the incidence of isolated CeCHT were similar in the UK to the Netherlands, we would expect to see 23–28 cases of CeCHT in England and Wales per year (9–12 with isolated TSH deficiency and 15–18 MPHD). However, whilst genetic testing may facilitate diagnosis as an adjunct to biochemical testing in isolated TSH deficiency, it will largely be negative in MPHD.

It is reasonable to assume that screening for CeCHT would facilitate early diagnosis and treatment, and available data suggest that there may be beneficial effects on neurodevelopment (35). However, the challenges of screening for CeCHT in the UK should not be underestimated. This would require redesign of the screening programme and workforce considerations for managing true and false screen-positive babies. Different worldwide screening strategies detect varying prevalence of CeCHT, which likely reflects differences in screening algorithms, including adjustment of TT4 cut-offs to balance risk of false positives and negatives (3, 17, 37). A formal health economic evaluation would be required to predict the financial costs and benefits of screening for CeCHT in the UK (2).

Further studies are required to elucidate the prevalence of CeCHT in the UK prospectively and the percentage of CeCHT cases with subnormal TT4 levels in newborn dried blood spot samples. Meanwhile, in the absence of biochemical newborn screening for CeCHT and with no timeline for routine national newborn genetic screening (if deemed acceptable by the public), there is a compelling need to invest in improving clinical diagnosis. Clinical initiatives are required to improve awareness of MPHD in non-endocrine and endocrine settings and to protocolize screening in high-risk settings (e.g. prolonged neonatal jaundice and recurrent hypoglycaemia) with comparison of thyroid biochemistry with bespoke, age-specific reference ranges. Parallel research initiatives are also required to determine novel biomarkers for CeCHT to enable it to be discriminated more robustly from other differential diagnoses, to maximize biochemical diagnosis.

## Supplementary materials



## Declaration of interest

NS was a member of the Scientific Advisory Bureau for Egetis Pharmaceuticals. CP, JL, DR, TC, CW, HH, CS and FA have no conflicts of interest that could be perceived as prejudicing the impartiality of the research reported.

## Funding

This research was supported by the Wellcome Trust (Senior Fellowship 219496/Z/19/Z to NS), the NIHR Cambridge Biomedical Research Centre (NS) and an NIHR Advanced Fellowship (CW).

## Data access statement

The dataset reported in this article is not available for sharing. Participants did not provide informed consent for broader data sharing, and further disclosure of the data could risk the identification of individual patients.
