# Supplementary material for: Retrospective, multicentre evaluation of central congenital hypothyroidism in the UK
Source: Eur Thyroid J. 2026 May 15;15(3):ETJ260014. doi: 10.1530/ETJ-26-0014 (PMC13193064; doi:10.1530/ETJ-26-0014)
Supplement: Supplementary file 1 [file supplementary_materials.pdf]

Supplementary Information.

Methods

Ethical approval

Initial data retrieval was performed at individual centres as part of trust-registered audits or service evaluation projects. Amalgamation of data and subsequent analyses were performed under the auspices of an ethically-approved project (REC 23/NE/0137) with approvals from local R&D departments. Pseudonymised databases from each institution were combined to form a single, linked-anonymised database by an independent administrator based at Newcastle upon Tyne Hospitals NHS Trust, with informed consent not required due to the preservation of anonymity and the non-interventional nature of the study.

Case Selection Criteria

Participating centres comprised four tertiary centres in England: Great Ormond Street Hospital, London (GOSH), Newcastle Hospitals NHS Foundation Trust, Newcastle upon Tyne (NH), Nottingham University Hospitals NHS Trust, Nottingham (NUH) and Cambridge University Hospitals NHS Foundation Trust, Cambridge (CUH). Each centre provides a paediatric endocrine service with expertise in managing CeCHT. Criteria and timeframes for case selection were centre-specific and tailored to local database availability and anticipated cohort size, aiming to include a representative cohort of cases from whom endocrine data was retrievable at diagnosis

Local case selection processes were as follows: CUH: a.) patients born between 2006 and 2021 with listed ICD codes for Central hypothyroidism, Congenital central hypothyroidism Secondary hypothyroidism, Hypopituitarism, Combined pituitary hormone deficiency Deficient secretion of one or more pituitary hormones, Deficient secretion of all pituitary

hormones. (n=450 cases) b.) patients on the paediatric GH (growth hormone) database (dating from 1994-2022), which contains details of all patients on GH treatment (n=55 cases). GOSH: a.) patient records were retrieved through electronic patient record search if at least one encounter at GOSH between April 2019 and Jan 2022, aged <16 years, with a coded diagnosis including any pituitary hormone deficiency and on thyroxine treatment. Patients with acquired hypopituitarism were excluded NH patients born between 2006 and 2021 with congenital central hypothyroidism and combined pituitary hormone deficiency on the local database. NUH a.) patients on a locally-maintained paediatric endocrine database born between 2000 and 2021 with a recorded diagnosis of hypothyroidism filtered by central causes, or with a diagnosis of combined pituitary hormone deficiency filtered by presence of TSH-deficiency.

Data collection

Clinical, radiological and biochemical data were retrieved retrospectively according to a standardized questionnaire by a local clinician with expertise in paediatric endocrinology. This captured the timeframe, clinical and biochemical pathway to diagnosis and treatment of CeCHT. Year of birth was recorded within a 2-year range to protect anonymity. Categorical responses were recorded as 'yes' or 'no' if there was robust supporting data. 'Not known' (NK) or 'not applicable' (NA) responses were amalgamated for analyses. Data were obtained from clinical letters and notes, with validation of biochemical data where possible and the best approximation recorded for dates where necessary.

The first abnormal thyroid function test was defined as the first result where either TSH or FT4 levels were outside the reported reference range, or, rarely, deemed to be abnormal in the clinical context (e.g. low normal FT4 in established or suspected pituitary pathology, due to additional pituitary hormone deficits and/or abnormal hypothalamo-pituitary axis on

imaging). Results at diagnosis were defined as those triggering commencement of levothyroxine. Time to treatment following first abnormal thyroid function tests provided an indicator of treatment delay. Additional pituitary hormone deficiencies were defined according to standard biochemical criteria or a requirement for long term hormone replacement. In males, the presence of genital indicators of hypogonadism (e.g. cryptorchidism, pathologically small phallus) were documented as indicators of gonadotrophin deficiency.

TSH and FT4 levels were expressed as absolute values and as standard deviation scores relative to the local reference range at the time of investigation (where SD score was calculated as $(x-\mu)/\sigma$, where μ is the mean of upper and lower limits of the reference range and σ is the difference of the upper and lower limits divided by 3.92). Cranial magnetic resonance imaging (MRI) or computerized tomography (CT) findings were classified according to the clinical radiology report. Since the quality of the report varied, imaging findings were classified as pituitary abnormality alone (e.g. ectopic posterior pituitary, anterior pituitary hypoplasia), Pituitary and extra-pituitary abnormality, Extra-pituitary abnormality alone, and normal imaging, and unknown finding (including cases who had not had cranial imaging). Extra-pituitary abnormalities included but were not limited to, defects of the cerebral hemispheres, including holoprosencephaly, or corpus callosum, and optic nerve abnormalities.

Neonatal manifestations were defined as those occurring within the first 28 days of life, with later features categorized as childhood manifestations. Neurodevelopmental sequelae were defined as objectively severe through the presence of an education, health and care plan (EHCP). The EHCP is a UK statement which formalizes the need for a tailored approach to

care and education due to neurodevelopmental or behavioural concerns. Potential confounders (NICU admission, preterm birth, comorbidities) were also recorded and height and weight Z-scores were computed from the UK 90 standards using the childsds package within R Statistical Software (v4.3.2; R Core Team 2023) (Vogel 2022).

Discussion

Naafs et al. (2020) report 148 cases of permanent central hypothyroidism in 3794207 screening participants (99.7% of 3805624), representing an incidence of 1:25637. Of these, n=60 (1:63237) had isolated TSH deficiency and n=94 (1:40364) had MPHD. In England and Wales, from 1995-2024 there were between 591072-729674 births each year. This gives an expected number of 23-28 cases of permanent central hypothyroidism (15-18 MPHD, 9-12 isolated TSH).

References

Naafs JC, Verkerk PH, Fliers E, *et al* Clinical and genetic characteristics of Dutch children with central congenital hypothyroidism, early detected by neonatal screening. *Eur J Endocrinol*. 2020 **183** 627-636.

Office for National Statistics (ONS), released 27 August 2025, ONS website, Dataset, Births in England and Wales: birth registrations.

Vogel M (2022). childsds: Data and Methods Around Reference Values in Pediatrics. R package version 0.8.0, <https://CRAN.R-project.org/package=childsds
